# Oral administration of hydrolyzed collagen alleviates pain and enhances functionality in knee osteoarthritis: Results from a randomized, double-blind, placebo-controlled study

**DOI:** 10.1016/j.conctc.2024.101424

**Published:** 2024-12-30

**Authors:** Juan Antonio Carrillo-Norte, Guillermo Gervasini-Rodríguez, María Ángeles Santiago-Triviño, Virginio García-López, Rafael Guerrero-Bonmatty

**Affiliations:** a Department of Medical and Surgical Therapeutics, Division of Clinical Pharmacology, School of Medicine, University of Extremadura, Badajoz, Spain; b Intensive Care Unit, University Hospital of Badajoz, Spain; c Department of Nursing, School of Nursing, University of Extremadura, Mérida, Spain

**Keywords:** Collagen peptides, Dietary supplement, Knee osteoarthritis, Joint pain, Joint functional limitation

## Abstract

Osteoarthritis (OA) is a major source of chronic pain and disability, representing a significant global health concern that affects 10–15 % of individuals aged over 60, with a higher prevalence among females than males. This investigation aimed to evaluate the impact of a dietary supplement containing collagen peptides (MW 1–3 kDa) on knee OA symptoms and inflammatory biomarkers such as C-reactive protein (CRP) and erythrocyte sedimentation rate (ESR). Adults aged 30–81 years (50 % female) with grade II or III OA and a minimum pain score of 40 on the 0 to 100 visual analogue scale (VAS) were enrolled. Participants were randomly assigned to receive either 10 g of the test product (verum group) or placebo and were assessed at baseline (T0, pre-treatment) and after a six-month follow-up period (T6). Baseline characteristics were comparable between groups. At T6, the verum group exhibited significant reductions in VAS pain scores, Lequesne algofunctional index (LAI) scores, CRP levels (mg/L), and ESR (mm/h) compared to placebo (p < 0.001). No adverse effects were reported during the study, and the supplement demonstrated good tolerability and yielded satisfactory safety and acceptability. These findings suggest that the dietary supplement may serve as a complement to drug therapy for knee OA by alleviating osteoarticular pain, improving locomotor function and potentially reducing reliance on analgesic and anti-inflammatory medications. This study provides valuable insights into the efficacy and safety of collagen peptides in managing knee OA symptoms.

## 1 Introduction

Osteoarthritis (OA) is a chronic, degenerative joint disorder characterized by the gradual breakdown and loss of hyaline cartilage, which primarily consists of type II collagen. This deterioration is accompanied by structural and functional alterations throughout the joint, such as synovial inflammation, the formation of bone osteophytes, and remodeling of the subchondral bone, where type I collagen is prevalent. Clinical symptoms encompass chronic joint pain, stiffness leading to reduced mobility or functional limitation, joint effusion, and varying levels of inflammation [[1], [2], [3]].

As life expectancy continues to rise, particularly in developed countries, OA has emerged as a primary contributor to chronic disability among the elderly population. It has a devastating impact on the patient's quality of life and the economic and social burden tied to OA are significantly high [[4], [5], [6]]. The knee is the most frequently affected joint in up to 10 % of men and 13 % of women over 60 years of age, with a progressive increase due to the aging of the population and other associated risk factors such as obesity and sedentary lifestyles [7,8].

Cartilage degradation alone does not solely dictate the severity of OA. Inflammation is now widely recognized as another significant aspect of OA, often persisting alongside cartilage degradation. Numerous studies have underscored a direct correlation between joint inflammation and the progression of the disease [2,[9], [10], [11]].

Various well-established factors, including pro-inflammatory cytokines such as interleukins (IL)-1β, IL-6, IL-8, and tumor necrosis factor-alpha (TNF-α), alongside pro-catabolic mediators acting through their respective signaling pathways, play crucial roles in OA. Furthermore, the impacts of nuclear factor kappa B (NFκB) and mitogen-activated protein (MAP) kinase signaling responses are extensively characterized. The phases of inflammatory activity can influence the extent of synovial hypertrophy, as well as the severity and progression of the disease [12].

Erythrocyte sedimentation rate (ESR) and serum C-reactive protein (CRP) levels are well-established inflammatory markers that remain elevated in persons with moderate to severe OA knee [13,14].

OA remains one of the few chronic aging disorders with limited effective treatment options, none of which have been demonstrated to delay disease progression [5]. The lack of disease-modifying therapies highlights a critical gap in knee OA management. Pharmacological interventions for knee OA, such as non-steroidal anti-inflammatory drugs (NSAIDs) and corticosteroids, primarily aim to alleviate pain and reduce inflammation [15].

While effective for symptomatic relief, these treatments often fail to address the underlying pathology of OA, which includes cartilage degradation, subchondral bone remodeling, and synovial inflammation. Furthermore, due to the chronic and progressive nature of OA, these medications are frequently prescribed for long-term use, which may exacerbate disease progression and lead to increased reliance on drugs, significantly heightening the risk of iatrogenic complications and adverse drug reactions [16,17].

The class of drugs known as Symptomatic Slow-Acting Drugs for Osteoarthritis (SYSADOA) includes agents like glucosamine sulfate and chondroitin sulfate. These compounds are widely recognized for their favourable safety profile and excellent tolerability among patients [18]. However, evidence supporting their efficacy as disease-modifying agents in the treatment of OA remains inconsistent and often unconvincing, as reported in numerous studies [[19], [20], [21], [22]].

These findings underscore the need for innovative therapeutic approaches that not only alleviate symptoms but also target the multifaceted pathology of OA.

In recent years, numerous studies have investigated the efficacy and safety of collagen-based nutraceutical supplements for OA management, as a potential therapeutic option [23].

Hydrolyzed collagen (HC) is a mixture of collagen peptides with a molecular weight between 1 and 3 kDa, that is obtained from the gelatinization and subsequent enzymatic hydrolysis of native collagen from animal tissues rich in this protein [24]. HC supplements are rich in the amino acids hydroxyproline, proline and glycine, with hydroxyproline being unique to collagen-derived products [25].

Several studies have shown that HC is enzymatically more easily absorbed, has higher bioavailability, is distributed to joint tissues and has analgesic and anti-inflammatory properties, consistently showing symptom-relieving effects, thus improving joint function and reducing joint pain [26], as well as optimizing the patient's quality of life [27].

Collagen supplementation aims to increase the anabolic response within damaged articular cartilage, potentially offering therapeutic benefits in OA management [23].

Various studies suggest that sustained daily intake of HC can alter the composition and concentration of hydroxyproline peptides in human blood [28] as well as modulate the activity of exo- or endo-type proteases in the digestive tract, which may contribute to the observed beneficial effects on articular cartilage. Specifically, collagen-derived peptides have been shown to accumulate within cartilage tissue, where they stimulate chondrocyte activity and promote the synthesis of extracellular matrix components, such as collagen and proteoglycans, which are essential for maintaining cartilage integrity and mitigating progressive tissue degeneration [29].

Moreover, a recent and comprehensive literature review highlights the diverse orthopedic benefits of collagen supplementation. These include increased bone strength, density, and mineral mass; reduced degradation of the extracellular matrix; suppression of inflammatory cytokines; improved joint stability, functional capacity, and mobility; enhanced muscle recovery; significant pain relief; and attenuation of biomarkers indicative of joint cartilage degradation [30].

Additionally, it has been shown that vitamin C exhibits chondroprotective properties and may play a therapeutic role in OA through stimulation of collagen synthesis, modulation of chondrocyte apoptosis and reduction of the activity of nuclear factor kappa B (Nf-κB) and matrix metalloproteinase (MMP-3) [31,32]. Notably, the inhibition of NF-kB signalling appears crucial in OA management, as it helps regulate inflammatory responses in macrophages [33]. Furthermore, selective inhibition of MMPs is considered a promising therapeutic strategy for the management of OA [34]. Vitamin C not only plays a role in collagen synthesis but also possesses antioxidant properties that may protect cartilage from oxidative stress, thereby promoting overall joint health [31]. Vitamin C deficiency, therefore, could be considered a risk factor for OA development, suggesting that its supplementation might represent a viable strategy for primary prevention. This approach may be particularly effective when combined with conventional or alternative therapies [35].

Given the substantial economic burden associated with OA, the use of a simple, cost-effective, and widely accessible supplement like hydrolyzed collagen warrants further exploration for its potential to mitigate the impact of this debilitating condition.

This study aims to evaluate the efficacy of a 6-month regimen of a dietary supplement containing collagen peptides with an average molecular weight of 1–3 kDa, combined with vitamin C (COLLinstant®), in alleviating pain and improving functional symptoms among individuals diagnosed with knee OA. Additionally, the study seeks to assess the safety, overall efficacy, and patient satisfaction with the product throughout the treatment and follow-up period.

## 2 Materials and methods

### 2.1 Clinical study design and ethical aspects

This investigation was conceptualised as a single-center, prospective, randomized, double-blind, placebo-controlled clinical trial conducted at GALA Laboratories (Don Benito-Villanueva, Spain) to evaluate the efficacy of COLLinstant® in alleviating symptoms of join-related pain and in improving joint mobility.

The investigation was performed according to the ethical guidelines detailed in the Declaration of Helsinki (amendment of the 64th General Assembly, Fortaleza, Brazil, October 2013) and with national regulations of Spain, and in full compliance with the applicable principles of good clinical practice (GCP) and International Council for Harmonisation (ICH) of Technical Requirements for Pharmaceuticals for Human Use [36]. The study protocol was reviewed and approved by the Clinical Research Ethics Committee at the University Hospital of Badajoz, Spain (code 20191120). This trial was registered at Clinical Trials.gov (Identifier: NCT05149053).

After written informed consent was obtained prior to any study procedures being initiated, patients were randomly assigned (1:1 ratio) to a strategy of receiving either COLLinstant® (verum group) or a placebo regimen (placebo group) by means of a computer-generated randomization list.

To guarantee the blindness of the physician and the participants, the sachets containing the investigational products were completely identical in appearance and the products were equal in flavor and texture.

**Fig. 1:**
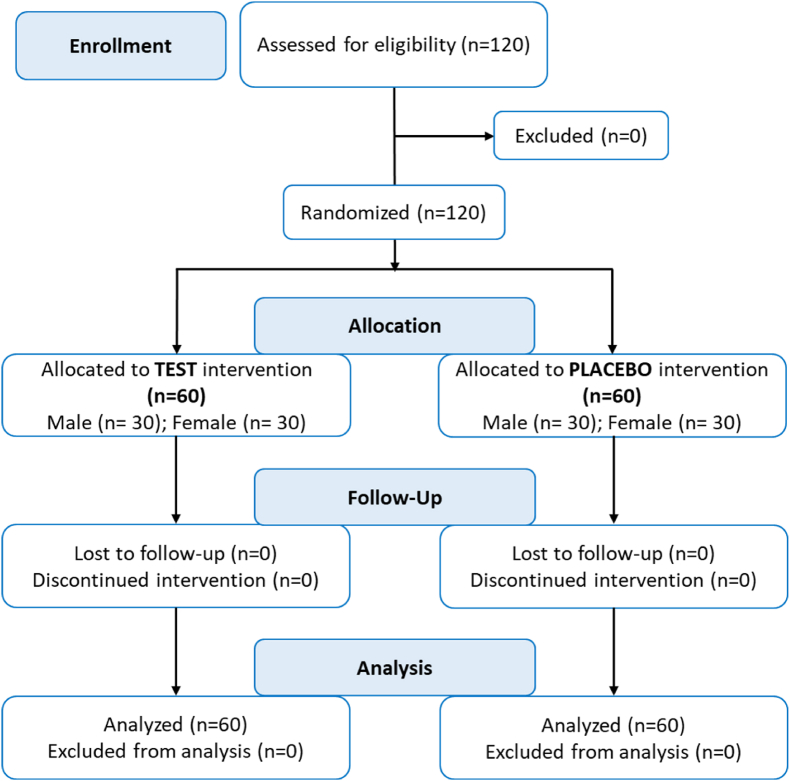
Recruitment of eligible patients with the intervention protocol and assessment.

For this purpose, the product was delivered in anonymous, coded, identical-looking boxes, so that both the patient and the investigators were unaware of the treatment assignment (double-blind). The identifying coding of the contents was performed by means of a bar code on the outer packaging. The data were not unblinded until data collection and database lock were both completed. Fig. 1 illustrates the process of recruiting and randomizing participants.

### 2.2 Study participants

Participants were recruited from the outpatient clinic of a physical medicine and rehabilitation department. All individulas underwent standard clinical and radiological evaluations and met the diagnostic American College of Rheumatology criteria for knee OA (unilateral/bilateral) [37]. Inclusion criteria were as follows: patients of both sexes aged ≥30 years; knee OA classified as grade II to III according to the Kellgren-Lawrence scale [38,39]; a knee joint VAS-pain score of ≥40 mm (out of 100 mm) lasting for more than 6 months, ensuring a homogeneous sample with moderate to severe knee OA symptoms. Participants were also required to be willing and able to comply with study instructions to use the test product, and complete the full study course; agree to maintain their usual diet and physical activity levels and to promptly report any changes in treatment regimen, clinical status, or laboratory results throughout the study; and agree to refrain from using any similar dietary supplements, glucosamine or chondroitin during the entire duration of the study.

Subjects were excluded in case of previous cardiovascular event in the last six months; history of liver or kidney disease; presence of a medical condition requiring chronic treatment with drugs or other substances; excessive alcohol consumption (>20g/day) or abuse of other substances; presence of intolerance or hypersensitivity to the food supplement or to any of its components in isolation; use of any intra-articular injection in the anatomical area under study in the last 6 months; treatment with SYSADOA in the last 3 months; criteria included in the Clinical Research Guide for medications used in the treatment of OA published by the European Medicines Agency of the European Union [40], and grade IV radiographic OA according to the Kellgren-Lawrence classification [38]; subjects cognitively impaired and/or unable to give informed consent; or had any other condition which in the medical investigator's opinion may adversely affect the individual's ability to complete the study or its measures or which may pose significant risk to the individual.

### 2.3 Investigational products

The test product is classified as a food supplement based on bovine bioactive hydrolyzed type I and III-collagen peptides with an average molecular weight between 1 and 3 kDa associated with vitamin C. Following ICH-GCP requirements and applicable local regulations [36], all investigational products were formulated as powder-containing sachets for a daily oral dose of 10.728 g.

Each sachet of the test product COLLinstant® (Viscofan DE GmbH, Weinheim, Germany) contained HC 10 g plus calcium ascorbate (vitamin C) 80 mg. The placebo was an identically appearing sachet that did not contain any nutrients. Other ingredients, which were also contained in the placebo, were 467 mg lemon flavour, 150 mg anhydrous citric acid, 8.5 mg sucralose and 7.1 mg stevia (97 %).

All patients (both verum and placebo groups) were instructed to dilute the content of one sachet (either the active supplement or placebo) in 200 ml of water, juice or another liquid and to consume it once daily, preferably in the morning, for six months.

Compliance, regarding the intake of the products was checked by the collection of unused supplements. All the product administered to the patients (food supplement and placebo) was manufactured by NutraResearch© SL (Barcelona, Spain).

### 2.4 Clinical endpoints

The study duration was six months. Measurements and assessments of clinical parameters were performed at baseline (T0) and after 6 months of intervention (T6).

The Visual Analog Scale (VAS) for pain assessment is a well-established, extensively utilized, and validated measure for both acute and chronic pain. Pain intensity was evaluated using a VAS ranging from 0 to 100 mm, with 0 indicating the absence of pain and 100 representing the highest imaginable pain intensity. Patients indicated their perceived level of pain intensity by marking a point on the scale.

To evaluate the algofunctional impact of gonarthrosis on the quality of life of the patient, the Lequesne algofunctional Index (LAI), a well-recognized questionnaire for its adequate validity, reliability and responsiveness was used [41]. It consists of an interview-format questionnaire, divided into three sections containing questions about pain intensity (five items), the distance that can be covered by the patient (one item) and how the activities of daily life are affected (four items). Each subscale can have a minimum score of 0 and a maximum score of 8, so the total score ranges from 0 (no pain or disability) to 24 (maximum pain and disability) [41]. To translate continuous measures from the numeric rating scale, into discrete categories, cut-off points are established. Thus, the variable is stratified into five categories of severity: mild (1–4), moderate (5–7), severe (8-10), very severe (11–13) and extremely severe (≥14).

Assessment of inflammatory activity was evaluated through determination of C-Reactive Protein (CRP) and Erythrocyte Sedimentation Rate (ESR) values. Thus, one nursing officer collected blood (fasting) (between 9 a.m. and 10 a.m.) from each person at baseline and at the final visit. The samples were then sent to the biochemistry laboratory. The blood samples were initially centrifuged at 4000 rpm for 5 min to obtain the clear super-natant serum. The serum samples were then preserved in a deep freezer (−80 °C). At a later date, the serum samples (in batches) were analyzed within three months of their collection.

The use of NSAIDs was thoroughly documented at baseline and closely monitored throughout the six months to ensure transparency and reliability in the findings and to evaluate the potential effects of collagen supplementation on overall medication requirements.

### 2.5 Safety and self-reported measures

Safety was assessed through the evaluation of adverse reactions reported by the patient that occurred during the treatment period. Adverse reactions were recorded at the final visit after six months of treatment.

At T6, after 6 months of treatment, the volunteers filled out questionnaires, to subjectively assess their perception of different parameters such as efficacy, organoleptic properties, tolerability and satisfaction since the last time they took the product. and a 3-point Likert scale with the following items: Dissatisfied, slightly satisfied and very satisfied, were used.

### 2.6 Statistical analyses

All statistical analyses were performed using IBM® SPSS® Statistics for Windows (version 27.0) and JASP [42]. The analysis of the distribution and normality tests of the variables were carried out using the Shapiro-Wilk tests.

All measured data are presented as mean ± SD (standard deviation), with the minimum and maximum values also provided to indicate dispersion. For categorical variables, the number and percentage of volunteers included in each category were calculated. The measured parameters were evaluated by descriptive analysis at T0 (baseline) and T6 (after 6 months of collagen supplementation). The efficacy was assessed based on relative changes in these parameters, calculated as the differences between means at T0 and T6.

Graphical representations of the results were generated using box-and-whisker plots. The box spans from the 25th to the 75th percentile of the data. Whiskers extend from the edges of the box to the minimum and maximum values that are not considered outliers. Outliers, defined as values more than 1.5 times the interquartile range from the box, are depicted as symbols beyond the whiskers.

The between-group comparison (placebo group vs. verum group) was carried out to determine population homogeneity at baseline (T0); and, to analyze treatment-related differences at T6, after 6 months of treatment, by means of the Mann-Whitney *U* test, a non-parametric test applied to two independent samples. Comparisons between categorical variables are performed using the Chi-square test.

The within-group mean changes of skin parameters between the initial and final visits (T0-T6) were evaluated by the non-parametric Wilcoxon signed-rank test, for paired data. Mean difference and 95 % confidence interval was provided. The threshold of statistical significance was set, in all cases, for a value of *p* < 0.05.

## 3 Results

### 3.1 CONSORT (consolidated standards of reporting trials) flowchart of the controlled interventional trial)

One hundred and twenty patients aged 30–81 years participated in our study and were randomized at baseline (T0) and allocated to the verum group (n = 60) or the placebo group (n = 60). No subjects had to be excluded during screening or throughout the study. No protocol violations occurred. Compliance during the trial was excellent thus, all volunteers (n = 120) who were screened for eligibility and requested to participate, completed the study protocol, and could therefore be analyzed. The flow of patients through the controlled intervention trial is depicted in a diagram according to CONSORT [43] (Fig. 1).

### 3.2 Characteristics of the population

The treatment groups (verum and placebo) were comparable at baseline (Table 1). All the volunteers were non-smoking patients. The demographic and baseline characteristics for the randomized study population are given in Table 1.

**Table 1:** Demographics and baseline data of the volunteers randomly allocated to the placebo and the test group.

	Placebo (n = 60)	Collagen (n = 60)	p value
**Age, years**	59.9 ± 9.9	59.2 ± 11.1	0.72^a^
**Sex, female/male, n (%)**	30/30 (50)	30/30 (50)	1.0^b^
**Height (m)**	1.68 ± 0.1	1.69 ± 0.1	0.68^a^
**Weight (kg)**	78.9 ± 9.9	79.1 ± 11.0	0.89^a^
**BMI (kg/m**^**2**^**)**	27.9 ± 3.7	27.7 ± 3.9	0.47^a^
**Pain VAS (0–100)**	63.4 ± 10.4	62.6 ± 6.3	0.10^a^
LAI score			
**Total score**	6.3 ± 1.3	6.4 ± 1.1	0.76^a^
Degree of disability, n, (%)			
Mild	4 (6.7)	1 (1.70)	
Moderate	50 (83.3)	52 (86.7)	0.59^b^
Severe	5 (8.3)	6 (10.0)	
Very severe	1 (1.7)	1 (1.7)	
**CRP (mg/L)**	2.8 ± 1.6	3.1 ± 1.4	0.11^a^
**ESR (mm/h)**	12.4 ± 4.5	13.8 ± 5.3	0.20^a^

Data are presented as means ± SD, except where otherwise indicated.

VAS: Visual Analogue Scale; LAI: Lequesne Algofunctional Index; CRP: C-Reactive Protein; ESR: Erythrocyte Sedimentation Rate.

a Mann-Whitney *U* test.

b Chi-square test.

### 3.3 Effect of the food supplement on pain

**Fig. 2:**
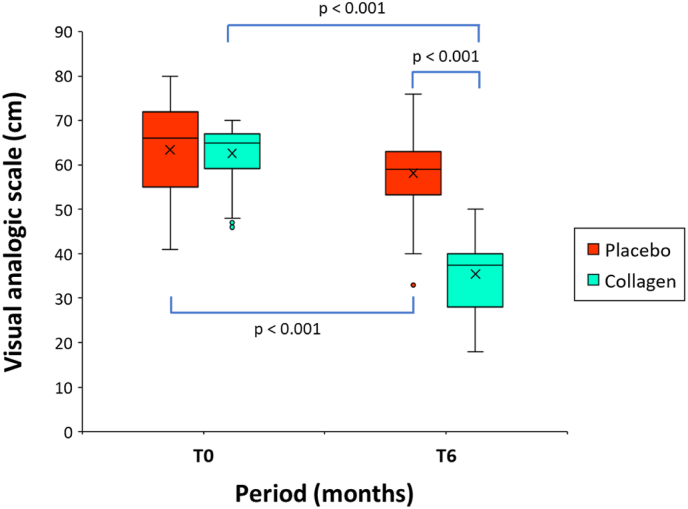
Boxplot of the 0 to 100 visual analogue scale (VAS) scores before (T0) and after intake of the investigational product for a 6-month period (T6) in the group of patients receiving a hydrolyzed collagen preparation or placebo (n = 60/group). The mean value (x) is also represented; p values indicate between-group (verum group vs placebo group; Mann-Whitney *U* test) and within-group (pre-treatment vs. post-treatment; Wilcoxon signed-rank test) statistically significant differences.

Fig. 2 shows the descriptive analysis of visual pain scale before intake of study products (at T0), and after six months of intake (at T6). At baseline, no significant differences were found for the mean pain scores between the placebo group and the verum group (Table 1).

The within-group (pre-treatment to post-treatment) difference in mean pain score was significantly decreased by −43.6 % (62.6 ± 6.3 vs. 35.5 ± 8.6 cm, mean difference, −27.2; 95 % CI, −25.3 to −29.1; p < 0.001), in the group of treatment with the investigational product. In the placebo group the percentage of change was lower and limited to −6.8 %.

At T6, the between-group difference (placebo group vs. collagen group) (58.1 ± 8.8 vs. 35.5 ± 8.6 cm, mean difference, −22.6; 95 % CI, −19.5 to −25.8; p < 0.001) as well as the difference between the relative changes in pain scores proves to be highly significant (p < 0.001) in favor of the test product. These results showed a significant improvement in the pain scores in the group of patients treated with the test product after six months of treatment.

At baseline, 43 (71.7 %) individuals within the placebo group and 17 (28.3 %) within the collagen cohort were utilizing analgesic or non-steroidal anti-inflammatory drugs (NSAIDs). However, by T6, the necessity for pharmacological intervention markedly decreased within the verum group, with only 2 (3.3 %) individuals requiring such treatment, in stark contrast to the placebo cohort where the proportion remained significantly higher at 25 (41.67 %) individuals (p < 0.001).

### 3.4 Effect of the food supplement on lequesne algofunctional index

**Fig. 3:**
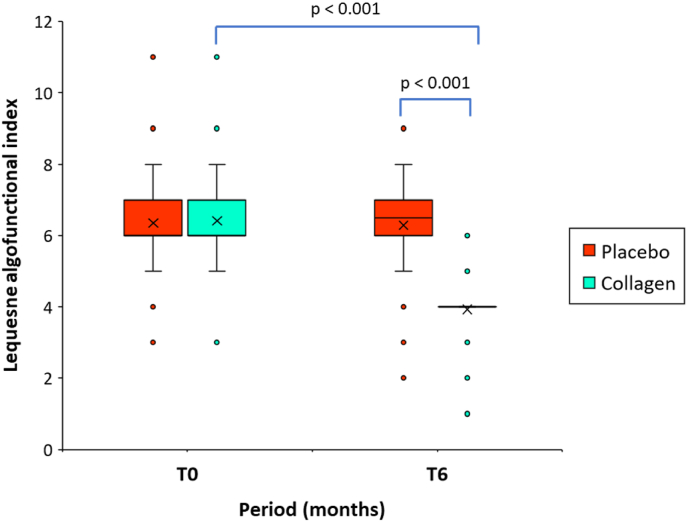
Boxplot representing Lequesne algofunctional index score at baseline (T0), and after intake of the product for a 6-month period (T6) in the group of patients receiving hydrolyzed collagen preparation, or placebo (n = 60/group). The mean value (x) is also represented; p values indicate between-group (Mann-Whitney *U* test) and within-group (Wilcoxon signed-rank test) statistically significant differences.

Fig. 3 shows the descriptive analysis of LAI at baseline (T0) and after six months of intake (at T6). At baseline, no significant differences were found for the mean scores between the placebo group and the verum group (Table 1).

The within-group (T0-T6) difference in the mean Lequesne algofunctional score was significantly decreased by −38.8 % (6.4 ± 1.1 vs. 3.9 ± 0.9; mean difference, −2.5; 95 % CI, −2.3 to −2.7; p < 0.001), in the verum group. In the placebo group the percentage of change was practically negligible and limited to −0.3 %.

At T6, the mean difference in the Lequesne algofunctional index was statistically significant between both groups (placebo group vs. collagen group) (6.3 ± 1.3 vs. 3.9 ± 0.8, p < 0.001). Moreover, the between-group difference in the relative changes from baseline in the Lequesne algofunctional scores proves to be highly significant in favor of the test product (p < 0.001).

Additionally, according to LAI scores, the percentage of patients included in each category varied from T0 to T6. After 6 months, the percentage of patients who presented a mild score on the LAI significantly increased (p < 0.001) in the group of patients treated with the test product. These results showed a significant improvement in the functional capacity, in the group of patients treated with the test product after six months of treatment. None patient was classified as extremely severe (Table 2).

**Table 2:** Evaluation of Lequesne Index scores measured at T6, following a 6-month treatment period with either a placebo or collagen supplementation. Population stratification based on disability was conducted using total scores obtained from a questionnaire assessing the impact of gonarthrosis on patient quality of life.

		Placebo (n = 60)	Collagen (n = 60)	P value
**Degree of disability, n, (%)**	Mild	5 (8.3)	54 (90.0)	
	Moderate	50 (83.3)	6 (10.0)	0.001^a^
	Severe	5 (8.3)	0 (0.0)	
	Very severe	0 (0.0)	0 (0.0)	

a Chi-square test.

### 3.5 Effect of the food supplement on C-reactive protein (CRP) concentrations

**Fig. 4:**
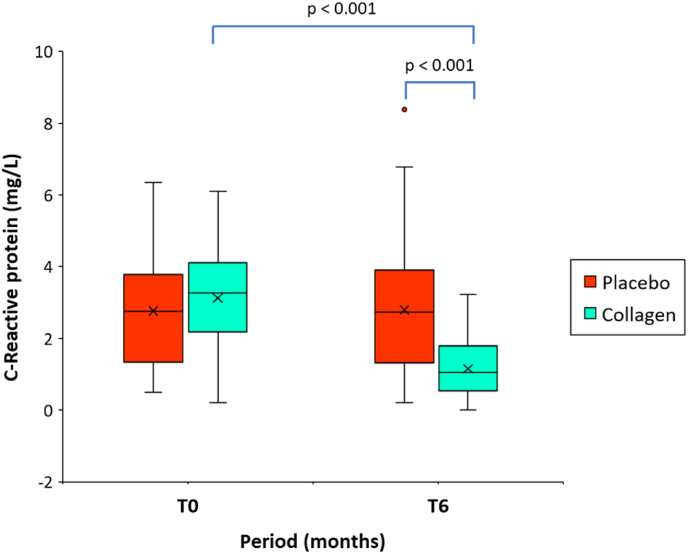
Boxplot representing C-reactive protein concentration (mg/L) at baseline (T0), and after intake of the study product for 6 months (T6) in the group of patients receiving hydrolyzed collagen peptides, or placebo (n = 60/group). The mean value (x) is also represented; p values indicate between-group (Mann-Whitney *U* test) and within-group (Wilcoxon signed-rank test) statistically significant differences.

Fig. 4 shows the descriptive analysis of C-reactive protein values before intake of study products (at T0) and after six months of intake (at T6). No significant differences were found for the mean initial C-reactive protein concentration at T0 for the placebo group and the group of patients taking the investigational product (Table 1).

The within-group (T0-T6) difference in the mean C-reactive protein concentration (3.12 ± 1.4 mg/L vs. 1.2 ± 0.8 mg/L; mean difference −1.9; 95 % CI, −1.6 to −2.3; p < 0.001) was significantly decreased by −56.2 %, in the verum group. On the contrary, the placebo group showed a percentage of change by 7.9 %.

At T6, when compared with the placebo group, the mean C-reactive protein concentration was significantly lower in the collagen group, (2.8 ± 1.6 mg/L vs 1.2 ± 0.8 mg/L, p < 0.001). The between-group difference (placebo group vs. collagen group) at T6, as well as the difference between the relative changes in C-reactive protein concentration proves to be highly significant (p < 0.001) in favor of the test product.

### 3.6 Effect of food supplement on erythrocyte sedimentation rate (ESR)

**Fig. 5:**
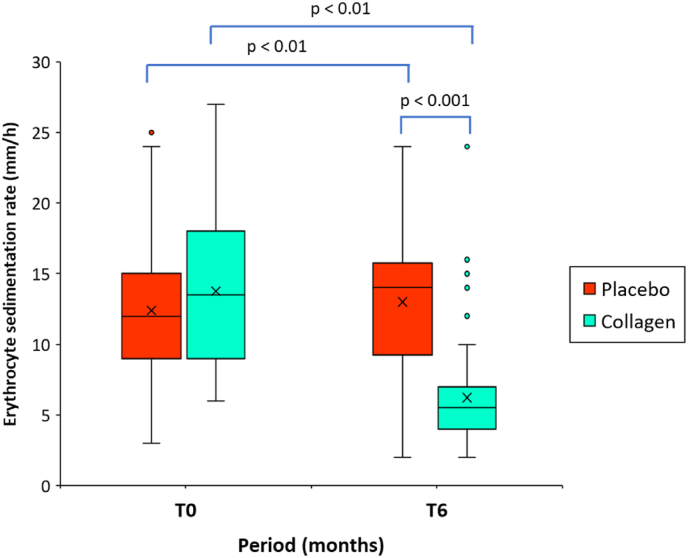
Boxplot representing erythrocyte sedimentation rate (mm/h) at baseline (T0), and after intake of the study product for 6 months (T6) in the group of patients receiving hydrolyzed collagen peptides, or placebo (n = 60/group). The mean value (x) is also represented; p values indicate between-group (Mann-Whitney *U* test) and within-group (Wilcoxon signed-rank test) statistically significant differences.

Fig. 5 shows the descriptive analysis of ESR (mm/h) values before intake of study products (at T0) and after six months of intake (at T6). No significant differences were for the mean initial ESR values at T0 for the placebo group and the verum group (Table 1).

The within-group (T0-T6) difference in the mean ESR was significantly reduced by −56.7 % in the group receiving the test product, with values of 13.8 ± 5.3 mm/h at baseline compared to 6.2 ± 4.0 mm/h at T6 (mean difference −7.5; 95 % CI, −6.7 to −8.4; p < 0.001). In contrast, the placebo group demonstrated a percentage change of 5.4 % and did not exhibit statistically significant within-group differences.

At T6, the mean ESR was significantly lower in the collagen group compared to the placebo group (13.0 ± 4.7 mm/h vs. 6.2 ± 4.0 mm/h, p < 0.001). The between-group difference at T6, as well as the difference between the relative changes in ESR from baseline, proved to be highly significant (p < 0.001) in favor of the test product.

### 3.7 Overall assessment of the food supplement efficacy

**Fig. 6:**
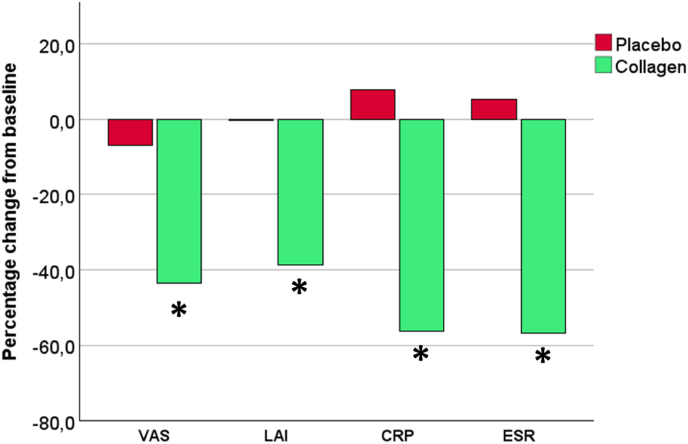
Percentage change of clinical parameters assessed in the study from baseline after 6 months of the interventional period (T0-T6) in the placebo group (red bars) and in the group of patients taking the investigational product (green bars). The asterisk (∗) indicates p < 0.001 for intergroup comparison (placebo group vs. collagen group; Mann-Whitney *U* test).

Fig. 6 summarizes the efficacy of the test product compared with placebo. The differences between the relative changes (T0-T6) in clinical parameters, namely the visual analogue scale, Lequesne algofunctional index, C-reactive protein, and erythrocyte sedimentation rate, are illustrated. Significantly improved clinical parameters were observed in the collagen group (p < 0.001).

### 3.8 Safety and subjective rating

No adverse events (AEs) were reported during the study. Compared with placebo, the percentage of patients who rated the satisfaction of the treatment as “good” was significantly higher in the group of patients taking the investigational product (93.4 % vs. 44.8 %, χ2 = 33.301; p < 0.001). The patients who rated the tolerability of treatment as “good” in the AGr was significantly higher in the group of patients taking the investigational product (100 % v. 67.2 %, χ2 = 20.931; p < 0.001). All patients in the test group rated the acceptability of the treatment as “Good” and had adequate therapeutic adherence.

## 4 Discussion

OA is a chronic condition characterized by a gradual and progressive worsening of clinical symptoms. Globally, it ranks among the top 50 most common sequelae of diseases and injuries, affecting more than 250 million individuals, which accounts for approximately 4 % of the world's population. Notably, knee OA represents the majority of the global disease burden for OA, comprising approximately 83 % of cases [39].

The central symptom is pain, which also has a negative impact on the patient's performance of daily activities. Therapeutic measures provide symptomatic relief and are theoretically aimed at delaying the progression of joint damage [44].

The updated guidelines from the American College of Rheumatology (ACR) and the American Academy of Orthopaedic Surgeons (AAOS) highlight the complexities of managing knee osteoarthritis (OA) [45,46]. Non-pharmacologic strategies, such as exercise, weight loss, and physical therapy, remain the cornerstone of treatment, providing modest improvements in pain and joint function. Commonly used medications for knee OA include over-the-counter pain relief medicines like acetaminophen and NSAIDs. These act as symptomatic treatment in the alleviation of pain, but the effect is temporary and have no effect on the disease process. NSAIDs, are recommended for short-term pain relief but are not suitable for long-term use due to risks such as nephrotoxicity, hepatotoxicity, and gastrointestinal complications [23].

Additionally, the class of dietary supplements known as SYSADOA is frequently prescribed and consumed with the aim of achieving disease modification. Among these, glucosamine and chondroitin sulfate are the most commonly used agents. However, several studies have reported contradictory results regarding their efficacy of these compounds as SYSADOAs and Disease-Modifying Osteoarthritis Drugs (DMOADs) [[19], [20], [21], [22]].

In contrast, dietary supplementation with hydrolyzed collagen has emerged as an alternative treatment and is gaining increasing attention as a promising strategy for the management of OA [[47], [48], [49]].

As an abundant extracellular matrix protein that supports cell attachment, function and survival, collagen plays an important role in the maintenance of healthy cartilage, bones and connective tissues. A loss of the protein due to advanced age, inflammatory processes, or excessive stress from factors like intense physical activity or obesity can result in joint pain, reduced mobility, and fragile bones.

While various formulations of collagen supplementation are available, collagen peptides seem to enhance collagen production in the body, demonstrating a positive effect on articular cartilage, tendons and ligaments, and significant therapeutic advantages for the management of OA [50,51].

The present placebo-controlled clinical trial was designed to analyze the efficacy of a food supplement containing standard molecular weight collagen peptides (1–3 kDa) on clinical signs and symptoms in patients with gonarthrosis. In addition, it has analyzed safety and the degree of acceptability of the treatment by patients.

In our study, the duration of treatment with collagen was similar to that of other studies, as was the methodology used to measure the effect of the food supplement on the variable pain and functional limitation in patients with gonarthrosis [52,53].

Recently, the efficacy of orally administered formulations of HC has been compared to intraarticular administration for managing symptoms of OA [54]. Given the necessity for frequent and controlled medication administration in treating this pathology, oral preparations are deemed more suitable [55]. Several studies have identified the ingested dose of HC as a determinant of its efficacy in conferring health benefits in knee OA [53].

In the study by Shigemura et al., effective doses to promote joint health benefit are based on an intake of HC greater than 153.8 mg/kg body weight, as a result of a significant increase in plasma hydroxyproline levels [28,29] equivalent to the dose of HC contained in the food supplement used in our study.

Other authors have obtained beneficial effects on the symptoms of gonarthrosis with lower doses of HC than those used in our study, but after intra-articular administration or by combining HC with different bioactive compounds such as chondroitin sulfate, glucosamine, L-carnitine, vitamins, and minerals [54,55].

Supplementation with antioxidant agents has become one of the most studied treatments in OA in the last decade. The investigational product administered contains, in addition to HC, vitamin C (80 mg), a compound that has shown encouraging results in terms of the prevention of OA, but not so much in the treatment. The concentration levels of antioxidant vitamins C (plasma) and E (serum) are significantly lower in patients with knee OA as compared with the control population [56]. Therefore, treatment with antioxidants in the initial stages of the disease may be useful as secondary therapy to attenuate the formation of reactive oxygen species in chondrocytes and prevent the oxidative damage and deterioration of the musculoskeletal tissues in OA [23,56]. Recently, several authors have provided evidence suggesting that vitamin C plays a pivotal role in orchestrating osteogenic differentiation and inhibiting osteoclastic activity [57,58].

Our findings reveal significant pain relief in patients with knee OA who received collagen peptides compared with those in the placebo group. The analgesic efficacy of collagen peptides in knee OA has also been demonstrated in several studies [49,59]. The within-group difference (T0-T6) observed in the mean scores obtained with the VAS scale was as high as 27 points (mm) in the verum group. In addition, this group exhibited a notable reduction by 43 % in pain intensity between the initial (pre-treatment) and final (post-treatment) visits (T0-T6).

Based on data collected from various meta-analyses, the minimum clinically important difference (MCID) for pain improvement in patients with knee OA has been estimated as a 2-point difference from baseline on the VAS (0-10) or a 10 %–20 % reduction in pain from baseline. For between-group comparisons (placebo group vs. experimental group), a range of MCID values from 10 to 19.9 points on the VAS (0–100) was considered [44].

According to our results, this allows us to affirm a priori that the decrease in pain intensity observed at the final visit of the study (T1) in the group receiving the supplement is not only statistically significant, but also clinically relevant, as it exceeds the established MCID thresholds.

Other authors have similarly suggested that patients who attain a score of less than 32.3 mm on the VAS (0–100) after their baseline visit achieve a clinically significant outcome [60], a finding closely aligned with the data obtained in our study.

On the contrary, other studies point out that the effect of collagen in the control of osteoarticular pain is not significant and there is insufficient evidence to make a recommendation for its widespread use in these patients [26,61].

There are no conclusive studies for the estimation of the MCID in the clinical course of the Lequesne index. When using this index to assess the long-term impact of active pharmacological treatment for OA of the hip, some studies have considered a minimal difference in the effect on the final score between 1.3 and 1.8 points [18,62].

In our study, the largest within-group changes were observed in the verum group. This group of patients who had taken collagen peptides for six months reported a significant improvement of 2.5 points (−39 %; p < 0.001) in LAI between the initial visit (T0) and the final visit (T1). Additionally, consistent with findings by other authors [52], the assessment of disability using the Lequesne Algofunctional Index (LAI) scores revealed a significant variation in the percentage of patients assigned to each category from baseline (T0) within the verum group. Notably, there was a significant increase (p < 0.001) in the percentage of patients presenting with mild LAI scores at T6 in the verum group compared with the placebo group.

Several clinical studies have evaluated the efficacy of HC in the treatment of OA, showing reductions in joint pain and improvement in function [63], indicating that HC has a positive therapeutic role in patients with OA.

The chondroprotective action of HC seems to be related to biological processes that stimulate the synthesis of elastin, type I and type II collagen and the formation of proteoglycans in the extracellular matrix of the chondrocytes [64].

Increased synthesis of extracellular macromolecules could reduce cartilage degradation, inhibiting proinflammatory processes and significantly reducing pain [26,65].

Some studies have observed that collagen supplementation demonstrates superior clinical efficacy and provides greater benefit compared to glucosamine sulfate in patients with knee OA [66,67].

On the other hand, erythrocyte sedimentation rate (ESR) and serum C-reactive protein (CRP) levels are well-established inflammatory biomarkers that can assess various aspects of the severity of the disease and can prove valuable in disease management and monitoring [14].

Patients exhibiting elevated CRP levels have heightened levels of cytokines and inflammatory alterations indicative of synovitis [68,69].

They are commonly used biomarkers in spondyloarthropathies and remain elevated in persons with moderate to severe OA of the knee [13,14]. A meta-analysis demonstrated higher hematological CRP concentration in OA patients compared to healthy volunteers [70]. It has been observed that OA knee patients experiencing greater pain tend to have higher CRP concentrations [68,71].

Consistent with findings from other studies, our results revealed significant reductions in hematological biomarkers, specifically CRP and ESR, following collagen supplementation [72]. In the verum group, mean CRP concentration and ESR levels decreased by 56 % and 57 %, respectively, indicating a substantial reduction in systemic inflammation. Furthermore, at the final assessment (T6), patients in the verum group exhibited significantly lower CRP concentrations and ESR values compared to those in the placebo group.

Nutraceuticals such as collagen peptides and vitamin C exhibit pleiotropic benefits, targeting multiple mechanisms such as pain alleviation, inflammation modulation, oxidative stress mitigation, and cartilage regeneration. Our findings reinforce this potential, as evidenced by the significant reduction in inflammatory biomarkers such as CRP and ESR [23,35,49].

By contrast, NSAIDs primarily offer symptomatic relief by reducing pain and inflammation but, they fail to address the underlying cartilage degeneration and other pathological mechanisms of OA.

These outcomes, combined with the observed reductions in the number of patients in the verum group requiring analgesics and/or NSAIDs, underscore the potential of collagen supplementation as promising complementary therapies in managing OA symptoms.

It is well-established that various factors, including medication use, dietary habits, and physical activity levels, can influence OA biomarkers [2]. To minimize these potential confounders, patients in our study were instructed to maintain their usual diet and lifestyle, while the use of SYSADOA was not allowed during the study.

In summary, a daily dietary supplement containing 10 g of collagen peptides (MW 1–3 kDa) demonstrated efficacy in alleviating osteoarticular pain and improving Lequesne index scores, indicating enhanced functional capacity in patients with knee OA following a six-month treatment period. Additionally, decreases in C-reactive protein (CRP) concentration and erythrocyte sedimentation rate (ESR) levels were observed. Patients also reported significant progress in performing daily activities and substantial improvement in quality of life, which contributed to a reduced reliance on analgesic and anti-inflammatory medications. This suggests a potential pathway to reduce both the dosage and frequency of drug administration while minimizing the risk of adverse effects associated with prolonged NSAID use. However, further clinical trials involving larger OA patient cohorts are warranted to confirm and expand upon these findings.

### 4.1 Limitations of the study

Certain authors have suggested potential alterations in cytokine levels associated with inflammation, including IL-6 and IL-10, within the synovial fluid of knee OA patients [53]. However, it is important to note that cytokine levels were not assessed in the current study. Furthermore, parameters such as diet, daily physical activity, and indirect factors like anxiety, stress, and cortisol levels can influence patients’ perception of pain. Nevertheless, these variables were not rigorously controlled for in this investigation.

## CRediT authorship contribution statement

**Juan Antonio Carrillo-Norte:** Writing – original draft, Validation, Supervision, Resources, Project administration, Methodology, Investigation, Formal analysis, Data curation, Conceptualization. **Guillermo Gervasini-Rodríguez:** Validation, Supervision, Methodology, Formal analysis, Conceptualization. **María Ángeles Santiago-Triviño:** Supervision, Resources, Methodology, Investigation. **Virginio García-López:** Supervision, Methodology, Investigation, Data curation. **Rafael Guerrero-Bonmatty:** Writing – original draft, Validation, Supervision, Resources, Methodology, Conceptualization.

## Declaration of competing interest

The authors declare that they have no known competing financial interests or personal relationships that could have appeared to influence the work reported in this paper.
